# Supplementary material for: Proximity coupling induced two dimensional magnetic order in EuO-based synthetic ferrimagnets
Source: Sci Rep. 2024 Sep 16;14:21586. doi: 10.1038/s41598-024-70548-7 (PMC11405716; doi:10.1038/s41598-024-70548-7)
Supplement: Supplementary file 1 — Supplementary Information. [file 41598_2024_70548_MOESM1_ESM.pdf]

Proximity Coupling Induced Two Dimensional Magnetic Order in EuO-Based Synthetic Ferrimagnets
# – SUPPLEMENTARY MATERIAL –

Paul Rosenberger

Fachbereich Physik, Universität Konstanz, 78457 Konstanz, Germany and

Fakultät Physik, Technische Universität Dortmund, 44221 Dortmund, Germany

Moumita Kundu, Ulrich Nowak, and Martina Müller*

Fachbereich Physik, Universität Konstanz, 78457 Konstanz, Germany

Andrei Gloskovskii and Christoph Schlueter

Deutsches Elektronen-Synchrotron DESY,

Notkestrasse 85, 22607 Hamburg, Germany

(Dated: August 12, 2024)

* martina.mueller@uni-konstanz.de

I. HELICITY-DEPENDENT HAXPES

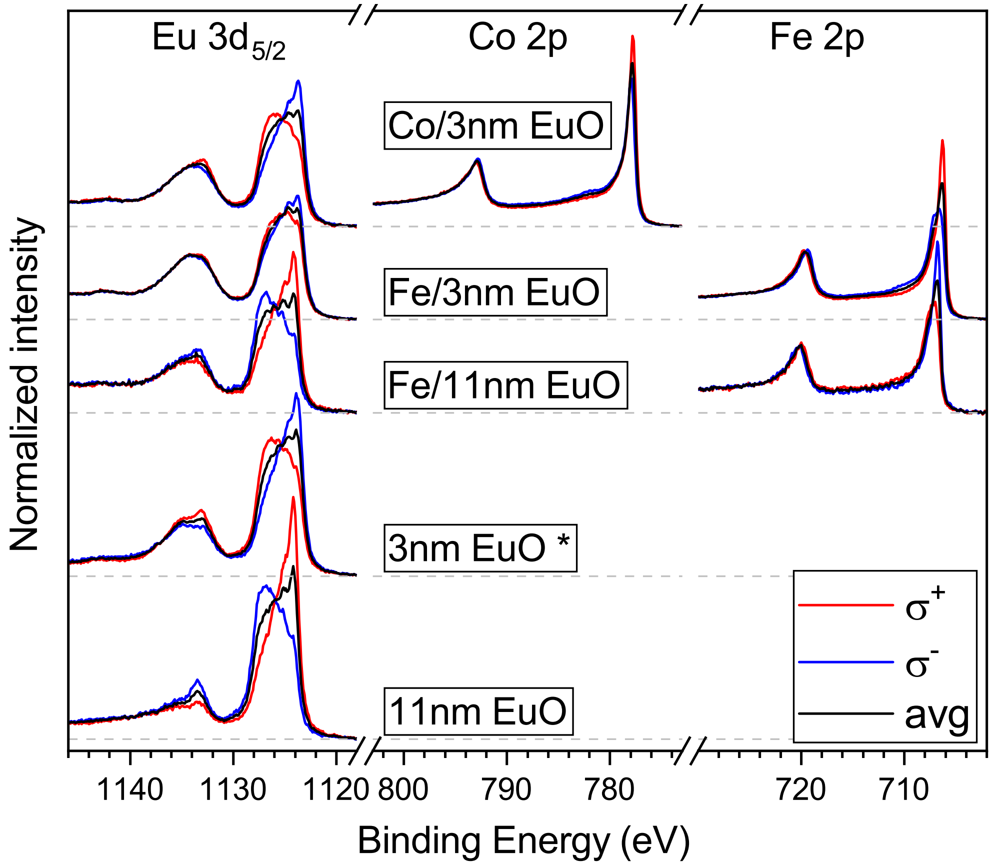

Supplementary Fig. 1. Helicity-dependent HAXPES in (close to) normal emission geometry of the Eu $3d_{5/2}$, the Co 2p and the Fe 2p core levels from the samples at T= 40K. The red and blue curves indicate the spectra obtained using light with σ^+ and σ^-, respectively. Crucial for our study is the stoichiometry of the EuO film. The intensity of the Eu^{2+}-satellite/Eu^{3+} peak in the binding energy range from 1130 eV to 1140 eV indicates that all samples are of good quality and therefore suited for our study. The 11nm EuO reference contains the lowest Eu^{3+} amount. The other samples have a similar Eu^{3+} content. Hence, the results of the angle-dependent measurements of all samples are comparable.

*: Note that the 3nm EuO reference sample was magnetized in the opposite direction than the other samples. for calculating the XMCD-PE, the spectra obtained with σ^+ and σ^- must therefore be interchanged.
